# Alkali treatment stabilizes fluctuations of urine AQP2 values measured by ELISA

**DOI:** 10.1007/s10157-015-1176-1

**Published:** 2015-10-13

**Authors:** Sei Sasaki, Yoko Saijo, Yasukazu Ohmoto, Fusako Iwata, Daisuke Koga, Kiyonori Katsuragi

**Affiliations:** Department of Nephrology, Tokyo Medical and Dental University, 1-5-45 Yushima, Bunkyo-ku, 113-8519 Tokyo, Japan; Diagnostic Division, Department of Research and Development, Otsuka Pharmaceutical Co., Ltd, Tokyo, Japan; Institute of Biomedical Innovation, Otsuka Pharmaceutical Co., Ltd, Tokyo, Japan

**Keywords:** Aquaporin-2, Exosome, Membrane protein, Water balance, Biomarker, Kidney

## Abstract

**Background:**

Aquaporin-2 (AQP2) in urine is now measured in many water-balance disorders and regarded as a useful biomarker for diagnosis and prognosis. An enzyme-linked immunosorbent assay (ELISA) method has been developed for measurement of large numbers of clinical samples. However, fluctuations in the measured values were sometimes observed depending on storage conditions. Urine AQP2 is present in exosome membranes and we speculated that this structural organization causes the fluctuations.

**Methods:**

Human urine samples from healthy subjects were measured by ELISA. Effects of maneuvers to disrupt the exosome membrane mechanically (freezing and thawing at different temperatures) and chemically (treating with alkali and detergents) prior to ELISA were examined.

**Results:**

Urine samples stored at 4 or −80 °C did not show significant AQP2 values, whereas those stored at −25 °C for more that 2 weeks provided the values. Urine samples treated with 0.4 N NaOH and 0.5 % Triton X-305 showed the consistent and comparable values to those stored at −25 °C.

**Conclusion:**

Pretreatment with alkali (0.4 N NaOH) to disrupt exosome membranes allowed consistent ELISA measurements of urinary AQP2. This simple method is applicable to ELISA of other membrane proteins included in exosomes.

## Introduction

Aquaporin-2 (AQP2) is a water channel protein in the kidney collecting duct that determines the urine concentrating ability of kidneys [1, 2]. AQP2, therefore, is deeply involved in water-balance disorders, and in turn, it could serve as a useful biomarker for diagnosis and prognosis of such diseases. Indeed, AQP2 was shown to be measurable in human urine soon after its cloning [3]. Since then, urine AQP2 has been measured in a wide variety of clinical disorders [2, 4, 5]. However, initially, it was a mystery how a membrane protein, like AQP2, could be excreted into the urine. In 2004, Pisitkun et al. reported that AQP2 is embedded in the membrane of exosomes in urine, resolving this dilemma [6].

Exosomes are late endosomes (multiple vesicular bodies)-derived nano (40–100 nm) vesicles with a lipid bilayer membrane that are excreted from numerous types of cells [7]. Exosomes are now attracting researchers’ intense interest because they carry microRNA, mRNA, and cytosolic and membrane proteins from their originating cells [8]. Accordingly, exosomes could provide biomarkers for clinical diseases [9]. Exosomes in the urine, specifically, has been shown to harbor many clinically useful biomarkers, including membrane proteins like AQP2 [5, 6, 10, 11]. In quantitative measurements by radioimmunoassay (RIA) or enzyme-linked immunosorbent assay (ELISA) of membrane proteins located in exosome membranes, antibody recognition domains of the proteins are critical. If recognition domains are inside exosomes, disruption or lysis of exosome membranes is necessary, as has been discussed recently [5]. In this context, it should be mentioned that good antibodies against human AQP2 recognize its C-terminus that is located inside exosomes [6].

Urine AQP2 has been measured by RIA, ELISA, and Western blotting [3, 12–16]. Among these, ELISA may be most suitable for clinical use because of its high sensitivity, ease of handling (no radioisotope) and high throughput for testing a large number of clinical samples. We have developed a sensitive ELISA system for detecting urine AQP2 [15], and the “freeze and thaw” method has been used to disrupt the exosome membranes. However, sometimes, fluctuations of measured values have been noticed depending on sample storage conditions. We speculated that this fluctuation was caused by insufficient disruption of the exosome membranes.

## Materials and methods

### ELISA and antibodies

The sandwich ELISA method and the antibodies were the same as reported previously (Human AQP2 ELISA kit, Otsuka Pharmaceutical Co., Ltd., Japan) [15]. Briefly, mouse monoclonal antibody and rabbit polyclonal antibody were raised against a recombinant thioredoxin-fused human AQP2 protein (45–271 amino acid). The monoclonal antibody was used to coat a 96-well microtiter plate to trap AQP2. Then, the trapped AQP2 was sandwiched with the polyclonal antibody, followed by colorimetric detection of a horse radish peroxidase-coupled reaction. Concentrations of human AQP2 in the samples were calculated from a standard curve of the same recombinant human AQP2.

### Human urine samples

This protocol was approved by the institutional ethical committee of the Research Division at Otsuka Pharmaceutical Co., Ltd. (120718) and all study participants gave written informed consent. Urine samples were obtained from healthy subjects who had no evidence of recent kidney or urinary tract disease. Urine samples were stored at 4, −25, or −80 °C until the assay. Data were shown as the mean ± SEM and the statistical analyses were performed using SAS software (R9.1, SAS Institute, Japan). The Pearson’s correlation coefficient was used to assess the correlation of AQP2 values with and without NaOH pretreatment. When the measured AQP2 values were lower than the detection limit (0.22 ng/ml), the value of the detection limit was used for analyses. *P* < 0.05 was considered significant.

## Results

### Fluctuations of urine AQP2 values at different storage temperatures and durations

**Fig. 1:**
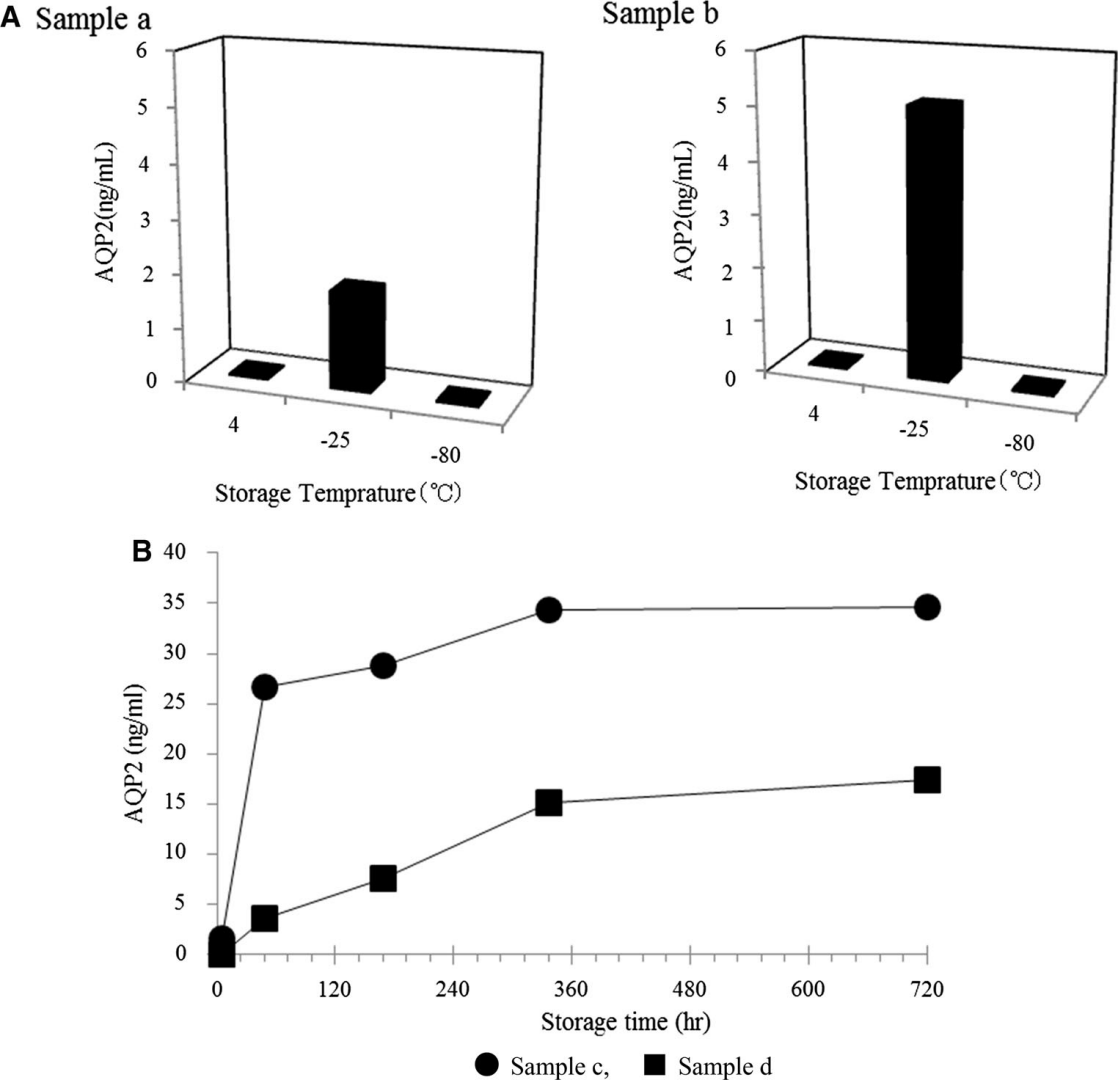
Effects of storage temperatures and durations. Two urine samples (sample *a* and *b*, **A**) were stored at 4, −25, and −80 °C for 1 day and AQP2 was measured. Only at −25 °C, AQP2 values were detectable. Another two different urine samples (sample *c* and *d*, **B**) were stored at −25 °C until one month and AQP2 were measured sequentially

Until measurements, clinical urine samples are usually stored in freezers with temperatures between −20 and −30 °C. Accordingly, we examined the effects of storage temperatures on ELISA results. Urine samples were stored at three different temperatures: 4, −25, and −80 °C for 1 day. AQP2 was detectable only in samples that were stored at −25 °C (Fig. 1A), and no AQP2 was detected in samples stored at either 4 or −80 °C. Furthermore, the measured AQP2 values increased during 2 weeks of storage at −25 °C (Fig. 1B). We interpreted these results as evidence that storage at 4 or −80 °C did not disrupt exosome membranes, while storage at −25 °C gradually disrupted exosome membranes and enabled the antibodies to contact to the intravesicular domains of AQP2 in our ELISA system.

To confirm the above results, urine samples were obtained from 18 subjects and stored at 4, –25, and −80 °C for 4 weeks. As summarized in Table 1, significant AQP2 values were measured in the samples stored at −25 °C, while almost zero values were detected in samples stored at 4 and −80 °C.

**Table 1:** Effects of storage temperatures of urine samples on AQP2 values

Temperatures (°C)	AQP2 concentrations (ng/ml)	Median (ng/ml)
4	0.22 + 0.00	0.22
−25	7.91 + 1.64	5.90
−80	1.00 + 0.52	0.22

Urine samples were obtained from 18 subjects and stored at 3 different temperatures for 4 weeks. Data are mean ± SEM

### Chemical treatment of urinary exosomes

**Fig. 2:**
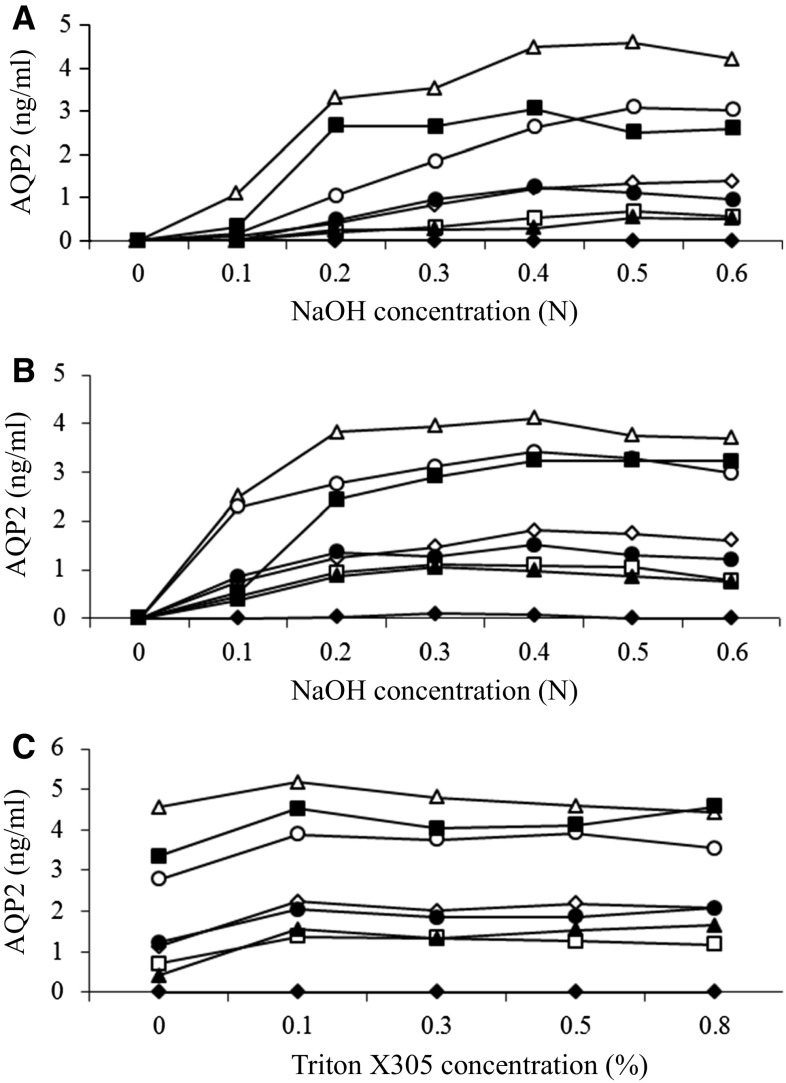
Effects of NaOH treatment and detergent. **A** Urine samples were treated with NaOH alone at different concentrations for 20 min. **B** Urine samples were treated with NaOH together with 0.5 % Triton X-305. **C** Urine samples were treated with different concentrations of Triton X-305 at fixed concentration of 0.4 N NaOH

Freezing at −25 °C for more than 2 weeks is time consuming and still leaves uncertainty. To overcome this problem, we examined chemical treatments of the exosome membranes. Because we observed in a preliminary study that detergents alone could not evoke AQP2 detection, we tried an alkali treatment (NaOH) with and without detergents. Urine samples (*n* = 8) were incubated with NaOH at different concentrations (0–0.6 N) for 20 min at room temperature, neutralized by HCl, and then tested by ELISA. In every sample, increasing NaOH concentrations up to 0.4 N increased the measured values of AQP2 even without a detergent (Triton X-305) (Fig. 2A), indicating the disruption of exosome membranes by alkali treatment alone. Addition of Triton X-305 at 0.5 % together with NaOH slightly increased and stabilized the AQP2 values (Fig. 2B). Similarly, at a fixed NaOH concentration of 0.4 N, increasing the concentration of Triton X-305 from 0 to 0.1 % increased measured AQP2 values and further increases up to 0.8 % showed no further increase or decline in AQP2 values (Fig. 2C). We additionally examined the effects of other detergents, Triton X-100, NP40, and Tween 20, but found that they were less effective than Triton X-305 (data not shown). We decided to use a combination of 0.4 N NaOH and 0.5 % Triton X-305 for 20 min as the pretreatment of urine samples prior to ELISA. The concentration of 0.5 % Triton X-305 was selected for safety in measuring urine samples that are highly concentrated or rich in protein content.

### Comparison of storage temperatures and the NaOH pretreatment

**Fig. 3:**
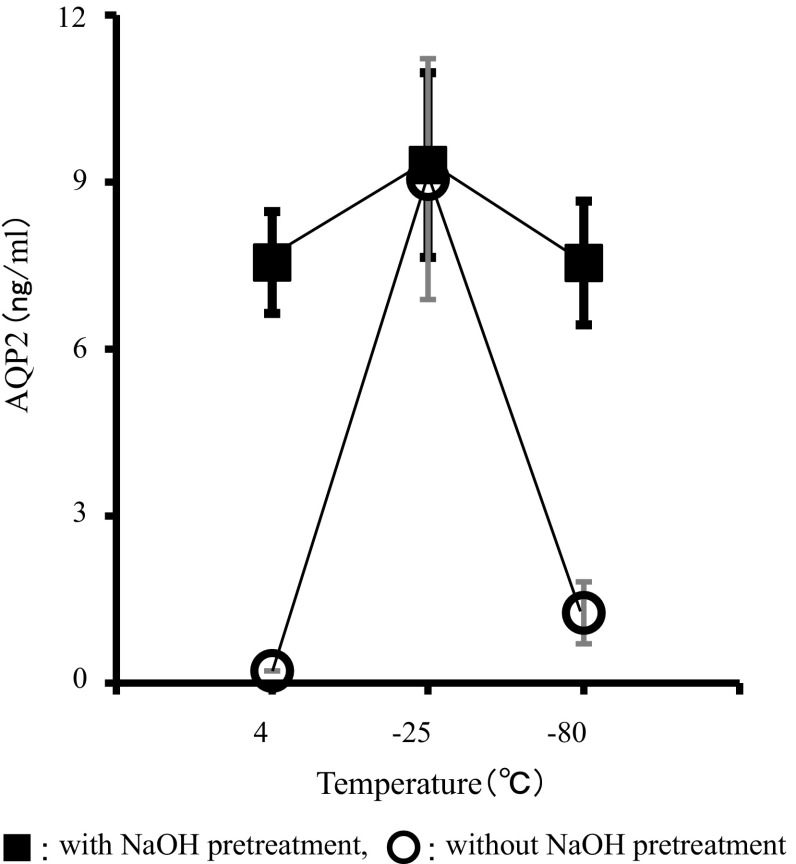
Comparison of storage temperatures and NaOH treatment in the same urine samples. Urine samples from 20 subjects were stored at 4, −25 , and −80 °C for 4 weeks, and AQP2 were measured with and without the pretreatment (0.4 N NaOH + 0.5 % Triton X-305). Data are shown as the mean ± SEM

To demonstrate the efficacy of the pretreatment with NaOH, urine samples from 20 subjects were stored at 4, −25, and −80 °C for 4 weeks, and AQP2 was measured by ELISA with and without the pretreatment (0.4 N NaOH + 0.5 % Triton X-305). Figure 3 clearly shows that without the pretreatment, AQP2 values were detectable only in those stored at −25 °C in every sample. On the other hand, after the pretreatment of NaOH, the same samples showed stable and comparable AQP2 values, irrespective of the storage temperatures (Fig. 3).

**Fig. 4:**
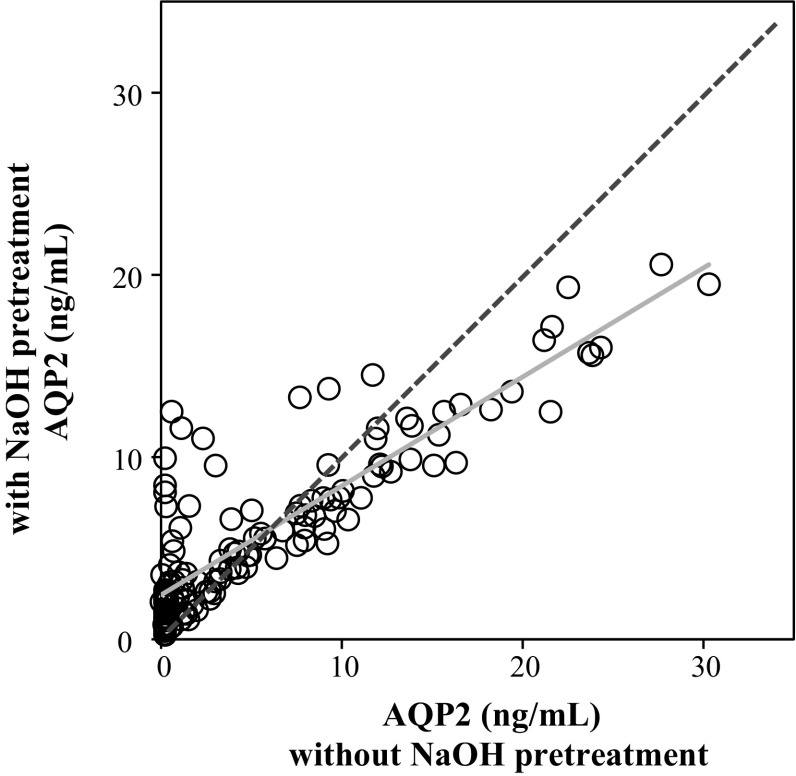
Comparison of AQP2 values with and without NaOH pretreatment. Urine samples stored at −25 °C for 2 weeks were measured with and without the NaOH pretreatment (0.4 N NaOH + 0.5 % Triton X-305), and the values were compared. A *solid line* shows the regression *line* of all data, *y* = 0.5984x + 2.4341 (*r* = 0.872, *p* ≤ 0.05). A *dotted line* shows a line of unity for a comparison purpose

In another study, urine samples stored at −25 °C for 2 weeks were measured with and without the alkali pretreatment, and the values were compared (Fig. 4). As a whole, the values with and without the pretreatment correlate well with a regression line of *y* = 0.5984*x* ± 2.4341 (*r* = 0.872, *p* ≤ 0.05, a solid line in Fig. 4). However, it is noteworthy that there are many points in which AQP2 values were very low without the pretreatment and became higher after the pretreatment (Fig. 4, a line of unity is shown in a dotted line for a comparison purpose). This result again indicates that storage at −25 °C sometimes is insufficient to disrupt the exosome membranes. Figure 4 also shows a tendency that in the range of high concentrations of AQP2, AQP2 values with pretreatment were smaller than the values without the treatment. This may reflect a possible loss of immunoreactivity of AQP2 by the alkali treatment, which would be theoretically inevitable, and we believe that the stabilizing effect of the alkali procedure is more important than its disadvantage of small loss of immunoreactivity.

## Discussion

It is important to know that the exosome lipid bilayer membrane is surprisingly strong and stable, and may not be disrupted even after 4 weeks of storage at 4 or −80 °C, as shown by the lack of AQP2 detection by our ELISA in samples without pretreatment (Table 1). Exosome membranes can protect their cargo for a long time in usual conditions. However, this favorable characteristic can inhibit immunological detection of antigens hidden in exosomes by blocking the access of antibodies inside exosomes. This consideration only applies to antibodies that recognize intracellular domains of membrane proteins of interest, as seen in our present case. It is worth noting that in exosomes, the inside corresponds to the inside of the cell, which is opposite of typical intracellular vesicles [6, 7].

In our previous attempts to measure urine AQP2 by RIA and ELISA [3, 12, 15], urine samples were stored in freezers (−25 °C), which could have resulted in the disruption of exosome membranes. However, as shown in Fig. 1, the measured values of urine AQP2 can change depending on freezing temperatures and durations. The storage at −80 °C, compared to −25 °C, seems to preserve exosome structures, because little AQP2 values were detected in −80 °C samples. The difference of effectiveness of the two temperatures (−25 vs. −80 °C) may indicate that freezing temperature is critically important and thawing itself is not so. Clearly, the fluctuations in measured AQP2 values depending on storage conditions should be eliminated before applying this ELISA method to a large number of clinical samples, and our efforts to solve this problem have led to the present results.

By incubating urine samples with a strong alkali prior to ELISA, measured AQP2 values became stable, irrespective of storage temperature and duration, indicating that the alkali treatment disrupted vesicle structure of exosomes (Fig. 3). The solubilizing ability of alkali allows it to be used with many substrates, such as lipids, peptides, and celluloses. Alkali has been applied to the isolation of a hepatitis B core protein from the virus core [17] and the recovery of functional proteins from herring [18]. This method is superior to the freeze and thaw method because of the short incubation time (20 min vs. 2 weeks) and consistency in ELISA results (see Fig. 3). Because the use of exosomes as potential resources for clinical biomarkers is receiving increased attention, this simple and convenient method for disrupting exosome membrane could be used in a wide range of applications.

It should be mentioned that measured values of urine exosome-derived biomarkers need to be adjusted (normalized) between samples. Several methods are available for this purpose such as timed urine collection, simultaneous measurements of urine creatinine, Tamm–Horsfall protein, or household exosome proteins [5]. For AQP2, normalization by simultaneously measured urine creatinine has been used in clinical spot urine samples [3, 12, 13, 15, 16], but we have to be careful whether this correction accurately estimates true excretion rate of AQP2 [19].

In conclusion, this study demonstrates that alkali treatment is valuable when proteins located in exosome membranes will be quantitatively measured by ELISA with intracellular domain-recognizing antibodies.
